# The relationship between Body Mass Index and Physical Fitness Index among college students: a cross-sectional study in Yangzhou, China

**DOI:** 10.3389/fpubh.2026.1780690

**Published:** 2026-02-25

**Authors:** Hailin Gong, Yuehui Zhao

**Affiliations:** College of Physical Education, Yangzhou University, Yangzhou, China

**Keywords:** Body Mass Index, college students, insufficient physical activity, obesity, Physical Fitness Index, physical health

## Abstract

**Background:**

Obesity and insufficient physical activity are major public health concerns among college students. Body Mass Index (BMI) and physical fitness levels are key indicators of functional health, but large-scale, objective measurement studies among Chinese college students suggest their correlation remains limited. This study aims to investigate the relationship between BMI and fitness index, and to explore gender differences.

**Methods:**

This study collected physical fitness test data from 28,861 undergraduate students in their first through fourth years at a university in Yangzhou, China, during the 2024 academic year. According to national standards, BMI is categorized into underweight, normal weight, overweight, and obese. Physical fitness is assessed using standardized tests covering lung capacity, flexibility, speed, explosive power, endurance, and muscular strength, with an overall fitness score calculated. The associations between BMI, gender, and physical fitness tests and scores were analyzed using two-way analysis of variance, correlation analysis, and quadratic regression models.

**Results:**

Body Mass Index is significantly correlated with most physical fitness test indicators. Students with normal BMI achieved higher overall fitness scores and better performance in most physical fitness tests, while underweight, overweight, and obese students performed worse. A higher BMI is associated with poorer performance in speed, endurance, and muscle strength, while lung capacity increases with rising BMI. Quadratic regression analyses revealed a significant nonlinear association between Body Mass Index and physical fitness, with optimal performance observed within the normal BMI range, and declines evident at both lower and higher BMI levels. This non-linear pattern differed by gender. Among males with higher Body Mass Index, the decline in physical fitness is more pronounced. Female students demonstrated significantly higher overall physical fitness levels than male students.

**Conclusion:**

Body Mass Index exhibits a distinct nonlinear relationship with physical fitness. College students with BMIs within the normal range demonstrate optimal physical fitness levels, while deviations from this range are associated with overall declines in physical fitness, particularly among male students. These findings provide a scientific basis for physical fitness monitoring and stratified intervention strategies for college students. However, due to the cross-sectional design of this study, causal relationships between BMI and physical fitness cannot be established, and future longitudinal studies are warranted.

## 1 Introduction

Obesity has become one of the most serious public health challenges of the 21st century, characterized by its widespread prevalence, rapid growth, and profound impact on the global disease burden (1). According to World Health Organization data, as of 2022, approximately 2.5 billion adults worldwide were overweight, with 890 million meeting the criteria for obesity (2, 3). This trend is projected to continue rising over the coming decades. Obesity is widely recognized as a major modifiable risk factor for multiple non-communicable diseases, including cardiovascular and cerebrovascular diseases, type 2 diabetes, and various malignancies (4, 5). It is also significantly associated with all-cause mortality, posing long-term challenges to public health systems and socioeconomic development (6).

Against this global backdrop, obesity among young people, particularly university students, has become an increasingly prominent issue (7, 8). The university years represent a critical transition period from adolescence to adulthood, serving as a crucial window for the formation and consolidation of healthy behaviors and lifestyles. However, multiple epidemiological surveys in recent years have shown that the obesity rate among Chinese college students has risen to 12.7%, with a noticeable trend toward younger age groups (9). This phenomenon reflects that obesity-related health risks are shifting to younger age groups at an earlier stage, necessitating urgent attention and intervention from a public health perspective.

Insufficient physical activity is considered one of the core behavioral factors driving the obesity epidemic (10). The World Health Organization notes that more than one-quarter of adults worldwide and approximately 80% of adolescents fail to meet the recommended levels of physical activity, with no significant improvement observed over the past two decades (11). Insufficient physical activity has been proven to be a major risk factor for cardiovascular disease, metabolic disorders, certain cancers, and various mental health issues (12–14). Currently, college students face increased academic pressure, widespread sedentary behavior, and insufficient participation in physical activities, making them a high-risk group for physical inactivity (15, 16).

Body Mass Index (BMI) is a widely used indicator for monitoring weight status in public health (17). Due to its simplicity in calculation and strong comparability, it is frequently employed for assessing health risks at the population level (18). Numerous studies indicate that BMI is significantly associated with the risk of developing various chronic diseases, and health risks markedly increase when BMI deviates from the normal range (19–21). Beyond the disease burden, abnormal BMI may also impact an individual’s physical function, quality of life, and mental health, further exacerbating public health risks (22, 23). Body composition assessments, such as dual-energy X-ray absorptiometry, bioelectrical impedance analysis, or skinfold measurements, can estimate fat and muscle composition more accurately than BMI (24, 25). However, the use of body composition measurements is often constrained by cost, technical requirements, and feasibility, particularly in large-scale population-based studies. From a public health perspective, BMI remains a practical and widely accepted proxy for weight status and obesity-related health risks.

Physical fitness levels serve as a crucial indicator of an individual’s functional health status, closely linked to disease prevention, healthy aging, and mortality risk (26, 27). The Physical Fitness Index (PFI) comprehensively reflects an individual’s cardiorespiratory endurance, strength, and adaptability to physical activity (28). Research indicates that there is a certain correlation between BMI and physical fitness, but this relationship is influenced by multiple factors such as gender, age, and social environment (29, 30). Among the specific population of college students in China, research systematically evaluating the relationship between BMI and physical fitness based on large-scale empirical data remains relatively scarce, and further evidence is urgently needed to supplement the existing body of work (31).

Based on this, this study utilizes physical fitness monitoring data from 28,861 undergraduate students (freshmen through seniors) at a university in Yangzhou, China during the 2024 academic year to systematically analyze the association between college students’ BMI levels and their physical fitness. Currently, issues such as insufficient participation in physical activities, rising obesity rates, and low cardiorespiratory fitness remain prevalent among college students. Physical education and health education still do not receive adequate attention in some higher education institutions. This study aims to provide scientific evidence for health monitoring, risk assessment, and intervention strategy development among university populations from a public health perspective, with the goal of promoting healthy lifestyles among college students and improving their long-term health outcomes.

## 2 Materials and methods

### 2.1 Participants

This cross-sectional study conducted a physical fitness survey among undergraduate students in their first through fourth years enrolled at a university in Yangzhou during the 2024 academic year. The study initially enrolled 29,143 students, of whom 84 were unable to complete the tests due to sudden physical conditions. An additional 67 students were excluded after preliminary screening identified them as having cardiovascular, respiratory, or metabolic diseases. A total of 28,992 students participated in the physical fitness assessment. During the testing process, data from 131 students was partially missing and was therefore excluded from the final analysis. Therefore, this study obtained complete data from a total of 28,861 participants. All participants signed informed consent forms, and the study protocol was approved by the Ethics Committee of Yangzhou University (Approval Number: YXYLL-2025-152).

### 2.2 Physical fitness test indicators

Our study followed the assessment protocol established in China’s National Student Physical Fitness Standards (2014 Edition). According to the 2014 standard version, the comprehensive physical fitness test encompasses multiple assessments. For males, these include BMI, lung capacity, sit-and-reach, 50-meter sprint, standing long jump, 1,000-meter run, and pull-ups. For females, they include BMI, lung capacity, sit-and-reach, 50-meter sprint, standing long jump, 800-meter run, and sit-ups. Physical fitness tests focus more on physical development functions and motor skills. BMI assesses body composition, lung capacity evaluates physical function, sit-and-reach measures flexibility, the 50-meter dash assesses speed, the standing long jump evaluates explosive power, the 1,000-meter and 800-meter runs assess endurance, and pull-ups and sit-ups evaluate muscular endurance. The testing methods and comprehensive score weightings for each project are shown in Table 1.

**Table 1:** The assessment plan in China’s National Student Physical Fitness Standards (2014 Edition) includes test items, testing methods, and item scoring weights.

Test	Unit	Evaluation	Testing method	Weight
BMI	kg/m^2^	Body shape	The formula for calculating BMI using height and weight is: BMI = weight (kg)/height^2^ (m^2^).	15%
Vital capacity	ml	Bodily functions	The test requires specialized equipment. The subject should stand or sit upright with feet flat on the ground and body relaxed. It is completed by taking a deep breath and then exhaling slowly.	15%
Sit-and-reach	cm	Flexibility	During testing, keep both legs straight with feet 10–15 centimeters apart. Use the middle fingers of both hands to push the cursor to its maximum range at a steady pace. Each participant is allowed two attempts.	10%
50 m run	s	Movement speed and reaction speed	Before the test, draw several straight 50-meter tracks on level ground. Designate one end as the starting line and the other as the finish line. Adopt a standing start position. Upon hearing the starting signal, immediately begin running and sprint toward the finish line with full effort.	20%
Standing long jump	cm	Explosiveness	The subject stands with feet shoulder-width apart behind the takeoff line, toes not touching the line. Both feet must take off simultaneously from the same spot without any shuffling or consecutive jumps. Each subject attempts three jumps, with the best single jump recorded.	10%
1,000 m run	min	Endurance quality	Test subjects must be grouped in pairs for testing, starting from a standing position. Upon hearing the command “Go,” subjects begin running. Timing starts when the flag is raised and stops when the subject’s torso crosses the vertical plane of the finish line.	20%
800 m run	min	Endurance quality		20%
Pull-up	times	Upper limb muscle strength and endurance	The subject jumps up and grips the bar with both hands in an overhand grip, arms straight and shoulder-width apart. After stabilizing, both arms simultaneously pull the body upward until the chin passes the top edge of the bar, completing one repetition.	10%
1 min sit-up	times	Core muscle endurance level	The subject lies supine on the mat with knees slightly bent and legs apart, fingers interlaced behind the head. The partner secures the lower limbs by pressing down on the ankles. One repetition is completed when the subject’s elbows touch or extend beyond the knees while sitting up. Record the number of repetitions completed within 1 min.	10%

### 2.3 Physical fitness test scoring

According to China’s National Student Physical Fitness Standards (2014 Edition), the BMI levels of college students of different genders are classified into four categories: underweight, normal weight, overweight, and obese. The BMI value ranges and scoring criteria for each category are shown in Table 2.

**Table 2:** BMI levels among college students by gender.

Level	Score	BMI (kg/m^2^)	
		Male	Female
Normal	100	17.9 ~ 23.9	17.2 ~ 23.9
Underweight	80	≤17.8	≤17.1
Overweight		24.0 ~ 27.9	24.0 ~ 27.9
Obesity	60	≥28.0	≥28.0

BMI, body mass index; BMI = weight (kg)/height (m)^2^.

According to China’s National Student Physical Fitness Standards (2014 Edition), the Body Mass Index (BMI) score is evaluated under a unified standard within the Physical Fitness Index for college students. However, the scores for physical function (vital capacity) and physical fitness (including sit-and-reach, 50-meter sprint, standing long jump, 1,000-meter run, 800-meter run, pull-ups, and sit-ups) are assessed under separate standards. Freshmen and sophomores are grouped under one standard, as shown in Table 3. Junior and senior students represent another standard, as shown in Table 4. These grade-specific scoring criteria are officially defined to account for age- and development-related differences in physical performance among college students.

**Table 3:** Physical fitness test scoring sheet for freshmen and sophomores.

Level	Score	Vital capacity (ml)		Sit-and-reach (cm)		50 m run (s)		Standing long jump (cm)		1,000 m run (min)	800 m run (min)	Pull-up (times)	1 min sit-up (times)
		Male	Female	Male	Female	Male	Female	Male	Female	Male	Female	Male	Female
Excellent	100	5,040	3,400	24.9	25.8	6.7	7.5	273	207	3′17″	3′18″	19	56
	95	4,920	3,350	23.1	24.0	6.8	7.6	268	201	3′22″	3′24″	18	54
	90	4,800	3,300	21.3	22.2	6.9	7.7	263	195	3′27″	3′30″	17	52
Good	85	4,550	3,150	19.5	20.6	7.0	8.0	256	188	3′34″	3′37″	16	49
	80	4,300	3,000	17.7	19.0	7.1	8.3	248	181	3′42″	3′44″	15	46
Pass	78	4,180	2,900	16.3	17.7	7.3	8.5	244	178	3′47″	3′49″		44
	76	4,060	2,800	14.9	16.4	7.5	8.7	240	175	3′52″	3′54″	14	42
	74	3,940	2,700	13.5	15.1	7.7	8.9	236	172	3′57″	3′59″		40
	72	3,820	2,600	12.1	13.8	7.9	9.1	232	169	4′02″	4′04″	13	38
	70	3,700	2,500	10.7	12.5	8.1	9.3	228	166	4′07″	4′09″		36
	68	3,580	2,400	9.3	11.2	8.3	9.5	224	163	4′12″	4′14″	12	34
	66	3,460	2,300	7.9	9.9	8.5	9.7	220	160	4′17″	4′19″		32
	64	3,340	2,200	6.5	8.6	8.7	9.9	216	157	4′22″	4′24″	11	30
	62	3,220	2,100	5.1	7.3	8.9	10.1	212	154	4′27″	4′29″		28
	60	3,100	2000	3.7	6.0	9.1	10.3	208	151	4′32″	4′34″	10	26
Fail	50	2,940	1960	2.7	5.2	9.3	10.5	203	146	4′52″	4′44″	9	24
	40	2,780	1920	1.7	4.4	9.5	10.7	198	141	5′12″	4′54″	8	22
	30	2,620	1880	0.7	3.6	9.7	10.9	193	136	5′32″	5′04″	7	20
	20	2,460	1840	−0.3	2.8	9.9	11.1	188	131	5′52″	5′14″	6	18
	10	2,300	1800	−1.3	2.0	10.1	11.3	183	126	6′12″	5′24″	5	16

**Table 4:** Physical fitness test scoring sheet for junior and senior students.

Level	Score	Vital capacity (ml)		Sit-and-reach (cm)		50 m run (s)		Standing long jump (cm)		1,000 m run (min)	800 m run (min)	Pull-up (times)	1 min sit-up (times)
		Male	Female	Male	Female	Male	Female	Male	Female	Male	Female	Male	Female
Excellent	100	5,140	3,450	25.1	26.3	6.6	7.4	275	208	3′15″	3′16″	20	57
	95	5,020	3,400	23.3	24.4	6.7	7.5	270	202	3′20″	3′22″	19	55
	90	4,900	3,350	21.5	22.4	6.8	7.6	265	196	3′25″	3′28″	18	53
Good	85	4,650	3,200	19.9	21.0	6.9	7.9	258	189	3′32″	3′35″	17	50
	80	4,400	3,050	18.2	19.5	7.0	8.2	250	182	3′40″	3′42″	16	47
Pass	78	4,280	2,950	16.8	18.2	7.2	8.4	246	179	3′45″	3′47″		45
	76	4,160	2,850	15.4	16.9	7.4	8.6	242	176	3′50″	3′52″	15	43
	74	4,040	2,750	14.0	15.6	7.6	8.8	238	173	3′55″	3′57″		41
	72	3,920	2,650	12.6	14.3	7.8	9.0	234	170	4′00″	4′02″	14	39
	70	3,800	2,550	11.2	13.0	8.0	9.2	230	167	4′05″	4′07″		37
	68	3,680	2,450	9.8	11.7	8.2	9.4	226	164	4′10″	4′12″	13	35
	66	3,560	2,350	8.4	10.4	8.4	9.6	222	161	4′15″	4′17″		33
	64	3,440	2,250	7.0	9.1	8.6	9.8	218	158	4′20″	4′22″	12	31
	62	3,320	2,150	5.6	7.8	8.8	10.0	214	155	4′25″	4′27″		29
	60	3,200	2050	4.2	6.5	9.0	10.2	210	152	4′30″	4′32″	11	27
Fail	50	3,030	2010	3.2	5.7	9.2	10.4	205	147	4′50″	4′42″	10	25
	40	2,860	1970	2.2	4.9	9.4	10.6	200	142	5′10″	4′52″	9	23
	30	2,690	1930	1.2	4.1	9.6	10.8	195	137	5′30″	5′02″	8	21
	20	2,520	1890	0.2	3.3	9.8	11.0	190	132	5′50″	5′12″	7	19
	10	2,350	1850	−0.8	2.5	10.0	11.2	185	127	6′10″	5′22″	6	17

Body Composition Score = (BMI Score × 15%), Physical Function Score = (Lung Capacity Score × 15%), Physical Fitness Score = (Sit-and-Reach Score × 10%) + (50-Meter Sprint Score × 20%) + (Standing Long Jump Score × 10%) + (1000-Meter/800-Meter Run Score × 20%) + (Pull-Up/Sit-Up Score × 10%). Total Score = Physical Morphology Score + Physical Function Score + Physical Fitness Score.

### 2.4 Statistical analysis

Descriptive statistics were calculated for all variables. A two-way analysis of covariance (ANCOVA) was performed to examine the interaction effects of BMI category and gender on physical fitness test scores, with grade (freshman, sophomore, junior, and senior) included as a categorical covariate. Analysis of covariance (ANCOVA) was also used to compare differences in physical fitness scores among different BMI categories and between genders while adjusting for grade. Using Spearman’s correlation analysis to explore the linear relationship between BMI and physical fitness test scores and physical fitness ratings. Use a quadratic regression model to analyze the nonlinear relationship between BMI and physical fitness scores. Statistical analysis was considered statistically significant at *p* < 0.05. All data analyses were performed using R statistical software version 4.5.1.

## 3 Results

### 3.1 Participant characteristics

The study included a total of 28,861 participants, comprising 14,485 men and 14,376 women. Participants were categorized into four groups based on their BMI levels: underweight, normal weight, overweight, and obese. The grade levels, body composition data, and physical fitness test results for all participants are compiled in the summary table, as shown in Table 5.

**Table 5:** Summary of descriptive characteristics for all participants.

Variables	All (*n* = 28,861)	Male (*n* = 14,485)				Female (*n* = 14,376)			
		Underweight (*n* = 361)	Normal (*n* = 8,892)	Overweight (*n* = 3,489)	Obesity (*n* = 1743)	Underweight (*n* = 593)	Normal (*n* = 11,782)	Overweight (*n* = 1,539)	Obesity (*n* = 462)
Grade [*n* (%)]									
Freshmen	7,809 (27.06%)	129 (35.73%)	2,188 (24.61%)	983 (28.17%)	605 (34.71%)	198 (33.39%)	2,987 (25.35%)	539 (35.02%)	180 (38.96%)
Sophomore	7,225 (25.03%)	118 (32.69%)	2,328 (26.18%)	779 (22.33%)	489 (28.06%)	205 (34.57%)	2,767 (23.49%)	407 (26.45%)	132 (28.57%)
Junior students	7,103 (24.61%)	67 (18.56%)	2,262 (25.44%)	875 (25.08%)	334 (19.16%)	99 (16.69%)	3,057 (25.95%)	324 (21.05%)	85 (18.4%)
Senior students	6,724 (23.3%)	47 (13.02%)	2,114 (23.77%)	852 (24.42%)	315 (18.07%)	91 (15.35%)	2,971 (25.21%)	269 (17.48%)	65 (14.07%)
Body shape data [*M* ± SD]									
Height (cm)	170.10 (8.49)	176.25 (6.10)				163.90 (5.54)			
		175.88 (5.91)	176.09 (5.95)	176.62 (6.00)	176.35 (6.98)	164.03 (6.11)	163.95 (5.40)	163.64 (5.52)	163.36 (7.76)
Weight (kg)	64.41 (14.19)	72.30 (13.91)				56.45 (9.13)			
		50.17 (4.70)	65.30 (7.30)	79.90 (6.54)	97.40 (12.19)	44.06 (4.32)	54.51 (5.90)	68.17 (5.36)	83.05 (11.25)
BMI (kg/m^2^)	22.13 (3.88)	23.24 (4.16)				21.00 (3.22)			
		16.21 (1.10)	21.03 (1.77)	25.58 (1.13)	31.31 (3.65)	16.35 (1.14)	20.25 (1.72)	25.43 (1.12)	31.24 (5.28)
Body function data [*M* ± SD]									
Vital capacity (ml)	3777.48 (1036.53)	4464.42 (834.29)				3085.32 (707.89)			
		3869.27 (720.63)	4370.17 (827.28)	4656.77 (811.48)	4683.53 (792.14)	2752.73 (624.37)	3084.39 (725.13)	3183.18 (604.08)	3210.05 (529.28)
Physical fitness data [*M* ± SD]									
Sit-and-reach (cm)	17.72 (7.20)	15.72 (7.51)				19.74 (6.27)			
		13.41 (7.93)	16.05 (7.57)	15.75 (7.21)	14.43 (7.51)	18.38 (6.08)	19.92 (6.28)	19.45 (6.15)	17.99 (6.22)
50 m run (s)	8.31 (1.09)	7.46 (0.62)				9.18 (0.71)			
		7.48 (0.54)	7.35 (0.58)	7.51 (0.61)	7.86 (0.70)	9.13 (0.67)	9.15 (0.70)	9.31 (0.73)	9.53 (0.83)
Standing long jump (cm)	196.14 (35.17)	223.02 (24.49)				169.05 (20.44)			
		224.16 (20.19)	226.94 (24.10)	219.81 (22.74)	209.21 (24.69)	169.26 (18.13)	169.86 (20.55)	164.63 (19.62)	162.95 (20.24)
1,000 m run (min)		4.25 (0.68)							
		4.23 (0.61)	4.14 (0.62)	4.30 (0.62)	4.70 (0.88)				
800 m run (min)						4.11 (0.56)			
						4.01 (0.55)	4.09 (0.55)	4.20 (0.54)	4.42 (0.64)
Pull-up (times)		5.21 (5.25)							
		6.20 (4.86)	6.20 (5.40)	4.05 (4.66)	2.30 (3.99)				
1 min sit-up (times)						39.04 (8.76)			
						40.13 (8.56)	39.50 (8.72)	36.56 (8.45)	34.37 (8.12)
Physical fitness test scores [*M* ± SD]									
Body shape score	13.92 (1.86)	13.48 (2.10)				14.36 (1.44)			
Body function score	12.15 (2.19)	10.39 (2.44)	11.83 (2.14)	12.62 (1.91)	12.72 (1.98)	10.91 (2.74)	12.18 (2.23)	12.66 (2.03)	12.83 (2.10)
Physical fitness score	45.67 (7.67)	43.54 (4.90)	44.87 (21.14)	41.72 (13.82)	36.43 (8.91)	49.21 (7.03)	48.64 (24.23)	46.68 (10.59)	43.85 (5.58)
Total score of physical fitness test	71.74 (8.81)	65.93 (7.52)	71.70 (8.00)	66.35 (7.50)	58.15 (8.74)	72.12 (6.79)	75.82 (6.80)	71.35 (6.96)	65.68 (7.72)

Physical morphology, physical function, flexibility, speed, explosive power, muscular endurance, and muscular strength indicators were measured for 28,861 participants. The average BMI of all participants was 22.13 with a standard deviation of 3.88. The rates of normal weight, underweight, overweight, and obesity were 71.63, 3.31, 17.42, and 7.64%, respectively. Among 14,485 male college students, the average BMI was 23.24 with a standard deviation of 4.16. The rates of normal weight, underweight, overweight, and obesity were 61.39, 2.49, 24.09, and 12.03%, respectively. The average BMI of 14,376 female college students was 21.00, with 81.96% having a normal weight—significantly higher than that of males.

Participants with BMIs in the obese range typically exhibit higher vital capacity, averaging 4683.53 milliliters for obese men and 3210.05 milliliters for obese women. Conversely, participants with a BMI in the underweight range scored lower on physical function tests. It is noteworthy that participants in the overweight and obese BMI ranges demonstrated slower performance in speed and endurance fitness measures—such as the 50-meter dash, 1,000-meter run, and 800-meter run—compared to participants of normal weight. The total score of the physical fitness test also decreased as BMI increased. Participants with normal weight had the highest average total fitness scores (71.70 for males, 75.82 for females), while the obese group had the lowest (58.15 for males, 65.68 for females).

### 3.2 Comparison of physical fitness tests across different BMIs and genders

**Figure 1:**
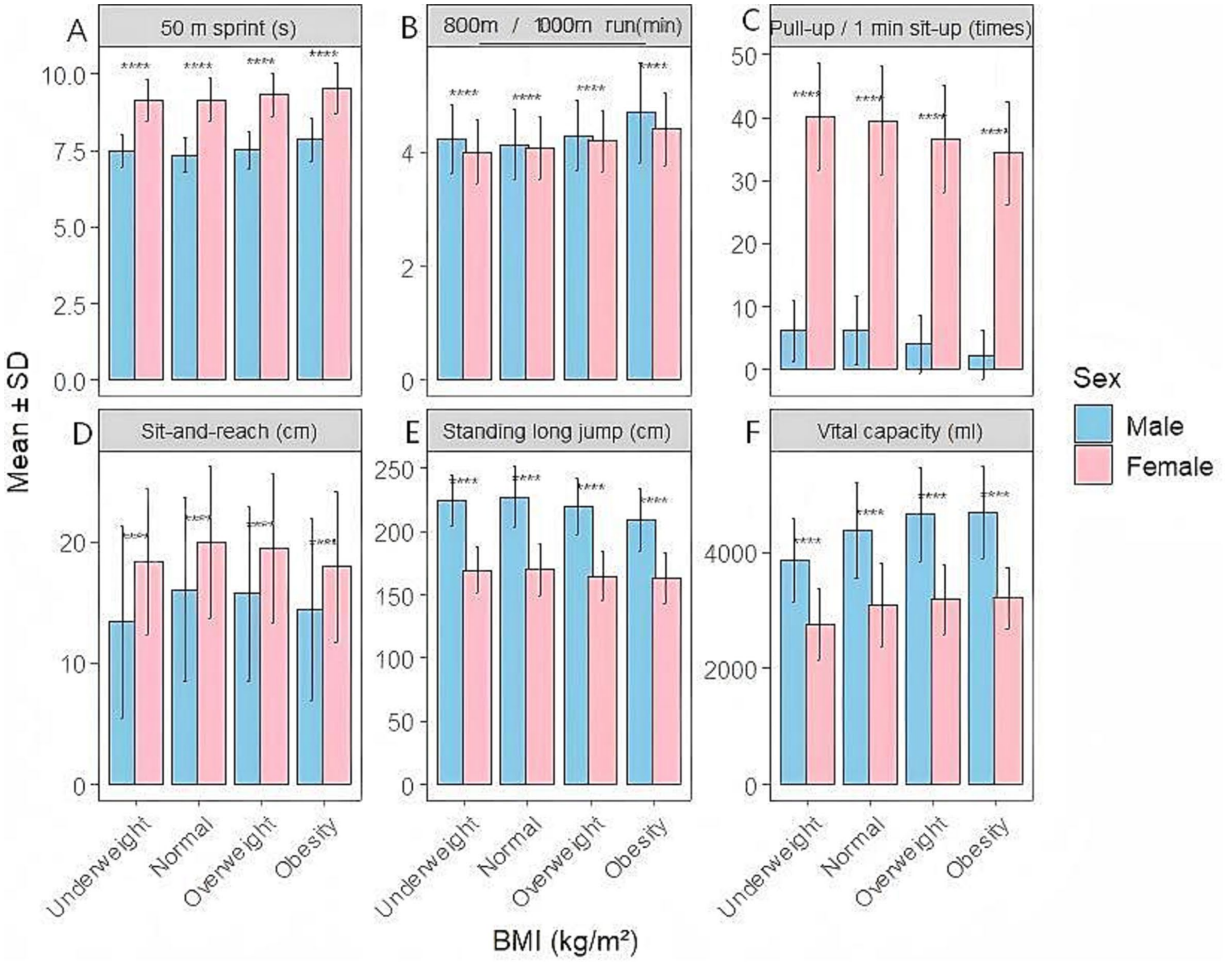
Comparative analysis of physical fitness tests across different BMIs and genders. **(A)** Comparison of 50-meter sprint times among college students of different BMI levels and genders. **(B)** Comparison of 800/1000-meter runs among college students of different BMI levels and genders. **(C)** Comparison of pull-ups/sit-ups per minute among college students of different BMI levels and genders. **(D)** Comparison of sit-and-reach flexibility among college students of different BMI levels and genders. **(E)** Comparison of standing long jump performance among college students at different BMI levels and of different genders. **(F)** Comparison of lung capacity among college students at different BMI levels and of different genders. When comparing the male and female groups, ****p < 0.001. BMI, body mass index; SD, standard deviation; cm, centimeter; kg, kilogram; m, meter; ml, milliliter; s, second.

A two-way analysis of covariance (ANCOVA) was conducted to examine the effects of gender and BMI levels on college students’ physical fitness indicators (Figure 1). After adjusting for grade, the main effects of BMI category and gender on physical fitness test scores remained statistically significant. The results indicate that gender and BMI exerted significant main effects on most physical fitness indicators (*p* < 0.05), with small to moderate effect sizes (gender: partial *η*^2^ = 0.04–0.21; BMI: partial *η*^2^ = 0.03–0.18) with some indicators showing clear interaction trends. Men significantly outperformed women in the 50-meter dash, standing long jump, lung capacity, and strength endurance tests (*p* < 0.001). Male college students demonstrate a particularly pronounced advantage in lung capacity and standing long jump performance. In comparison, female college students demonstrated significantly higher scores than males in the sit-and-reach test (*p* < 0.001), indicating superior flexibility levels. Overall, the normal weight group performed best in most physical fitness tests. In the 800/1000-meter run events, completion times significantly increased with rising BMI, with overweight and obese groups demonstrating markedly poorer performance than the normal-weight group (*p* < 0.001), Male college students exhibited moderately large effect sizes (Cohen’s *d* = 0.784–0.889). Female college students demonstrated moderate effect sizes (Cohen’s *d* = 0.503–0.689).

In the standing long jump and strength endurance events, the obese group performed significantly worse than other BMI groups, suggesting that excessive weight has a pronounced detrimental effect on explosive power and relative strength. In the seated forward bend test, BMI had a relatively minor impact on performance, but the overall flexibility level of the obese group remained lower than that of the normal-weight group (*p* < 0.05). Significant differences in vital capacity were observed across different BMI groups, with higher values in the normal-weight and overweight groups and relatively lower values in the underweight group (*p* < 0.05). The trend in mean changes indicates that the impact of BMI on physical fitness levels differs between genders. In men, the decline in endurance, explosive power, and strength endurance is more pronounced during overweight and obese states, while women exhibit relatively stable physical fitness changes across different BMI groups.

### 3.3 Comparison of physical fitness scores across different BMIs and genders

**Figure 2:**
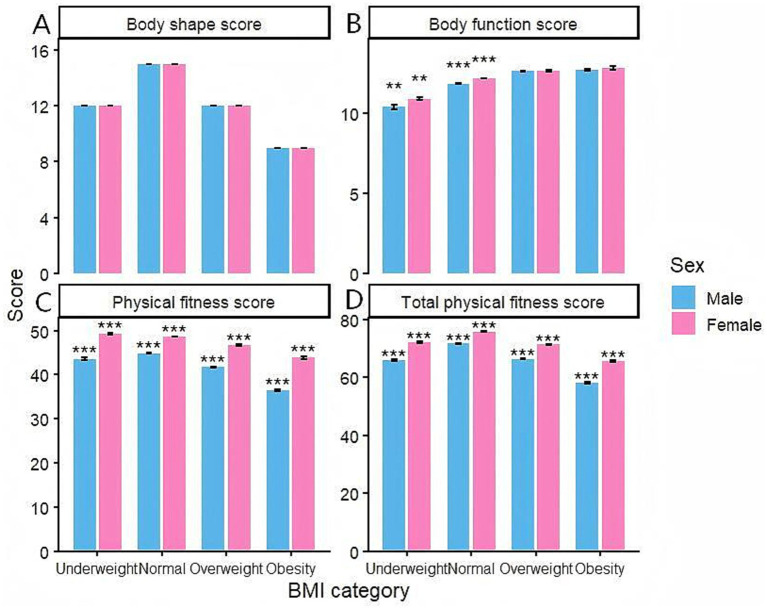
Comparison of physical fitness scores across different BMIs and genders. **(A)** Comparison of body shape scores among college students across different BMI levels and genders. **(B)** Comparison of physical fitness scores among college students across different BMI levels and genders. **(C)** Comparison of physical fitness scores among college students at different BMI levels and of different genders. **(D)** Comparison of total fitness test scores among college students across different BMI levels and genders. Note: When comparing the male and female groups, **(*p* < 0.05), ***(*p* < 0.01).

As shown in Figure 2, college students with different BMI levels and genders exhibit varying degrees of differences in body shape, physical function, physical fitness, and overall physical fitness scores. In terms of body shape scores, the normal weight group scored highest, while the obese group scored lowest. Overall, higher BMI correlated with lower body shape scores. In terms of physical function scores, the scores showed a gradual upward trend with increasing BMI levels, with the obese group scoring significantly higher than the underweight group (*p* < 0.05). This suggests that higher body weight may be more conducive to the performance of cardiopulmonary function or baseline functional capacity. Significant differences were observed in physical fitness scores across different BMI groups (*p* < 0.01), with the normal weight group scoring highest and the obese group scoring lowest. The normal weight group scored significantly higher than other groups in overall physical fitness scores (*p* < 0.01), indicating that college students within the normal BMI range demonstrated the highest level of comprehensive physical fitness. Girls generally outperformed boys in the physical fitness test. In terms of physical function scores, physical fitness scores, and overall physical fitness scores, female participants scored significantly higher than male participants (*p* < 0.01). This difference was particularly pronounced in both the normal weight group and the overweight group.

### 3.4 Correlation analysis between physical fitness tests and physical fitness scores across different BMIs and genders

**Figure 3:**
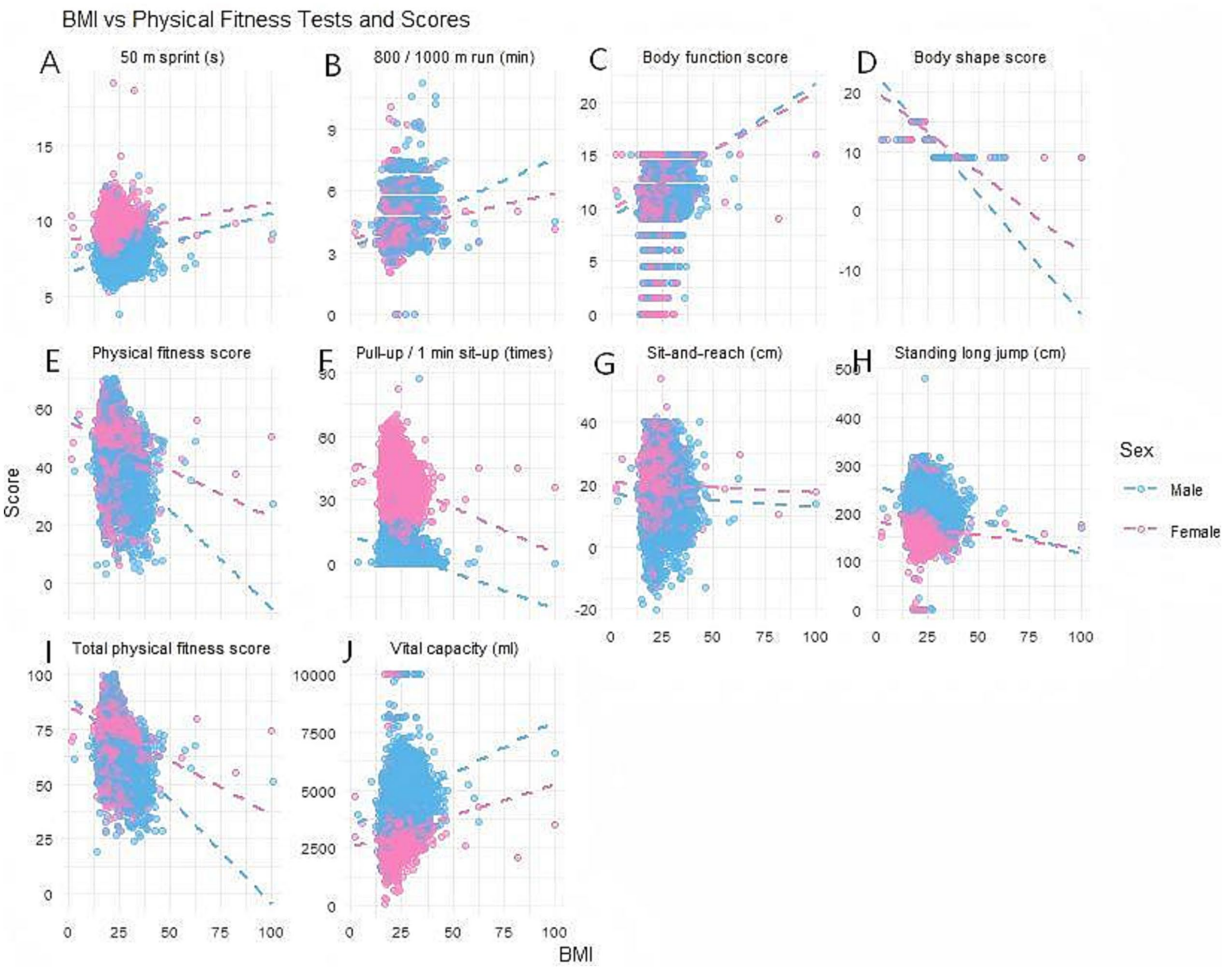
Correlation analysis between physical fitness tests and physical fitness scores across different BMIs and genders. **(A)** Correlation analysis of 50-meter sprint performance among college students across different BMI levels and genders. **(B)** Correlation analysis of 800/1000-meter runs among college students of different BMI levels and genders. **(C)** Correlation analysis of physical fitness scores among college students at different BMI levels and of different genders. **(D)** Correlation analysis of body shape scores among college students at different BMI levels and of different genders. **(E)** Correlation analysis of physical fitness scores among college students at different BMI levels and of different genders. **(F)** Correlation analysis of pull-ups/1-min sit-ups among college students at different BMI levels and of different genders. **(G)** Correlation analysis of sit-and-reach flexibility among college students across different BMI levels and genders. **(H)** Correlation analysis of standing long jump performance among college students at different BMI levels and of different genders. **(I)** Correlation analysis of total physical fitness scores among college students at different BMI levels and of different genders. **(J)** Correlation analysis of lung capacity among college students at different BMI levels and of different genders.

The concentration of scatter points and the slope of the trend line in Figure 3 visually reflect the strength and direction of the correlation. The 50-meter sprint and 800-meter/1000-meter run show a significant positive correlation of moderate strength (*r* = 0.42–0.58) with BMI, meaning that as BMI increases, the time required to complete the tests also increases. Given the large sample size, the magnitude of correlation coefficients suggests robust and practically meaningful associations, rather than merely statistical significance. The scattered points are closely clustered along the trend line, indicating that this correlation exhibits strong stability and statistical significance. Pull-ups/1-min sit-ups and standing long jump demonstrate a significant negative correlation with BMI, meaning higher BMI correlates with lower strength and power performance. The scatter plot shows a high degree of alignment with the trend line, further indicating that such correlations are consistent across different individuals. The scatter plot for the sit-and-reach test shows a relatively dispersed distribution, with the trend line approaching horizontal, indicating a weak correlation with BMI. Lung capacity is positively correlated with BMI; the higher the BMI, the greater the lung capacity.

The blue and pink scatter points in the figure represent male and female data, respectively, with their divergent trends reflecting significant gender heterogeneity. Women demonstrated greater sensitivity to BMI across most metrics. In indicators such as the 50-meter sprint, 800-meter run, physical fitness scores, and total scores, female data points exhibited significantly higher alignment with the trend line compared to males. This indicates a stronger negative correlation between female physical performance and BMI in these areas. Key research findings: Body shape scores, overall fitness scores, and total scores all exhibit a stable, strong negative correlation with BMI. That is, the higher the BMI, the lower the scores across all categories. The scattered points are concentrated and show consistent trends, indicating that BMI’s negative impact on overall fitness levels exhibits cross-dimensional stability.

### 3.5 Regression analysis of BMI and physical fitness scores across different BMI categories and genders

**Figure 4:**
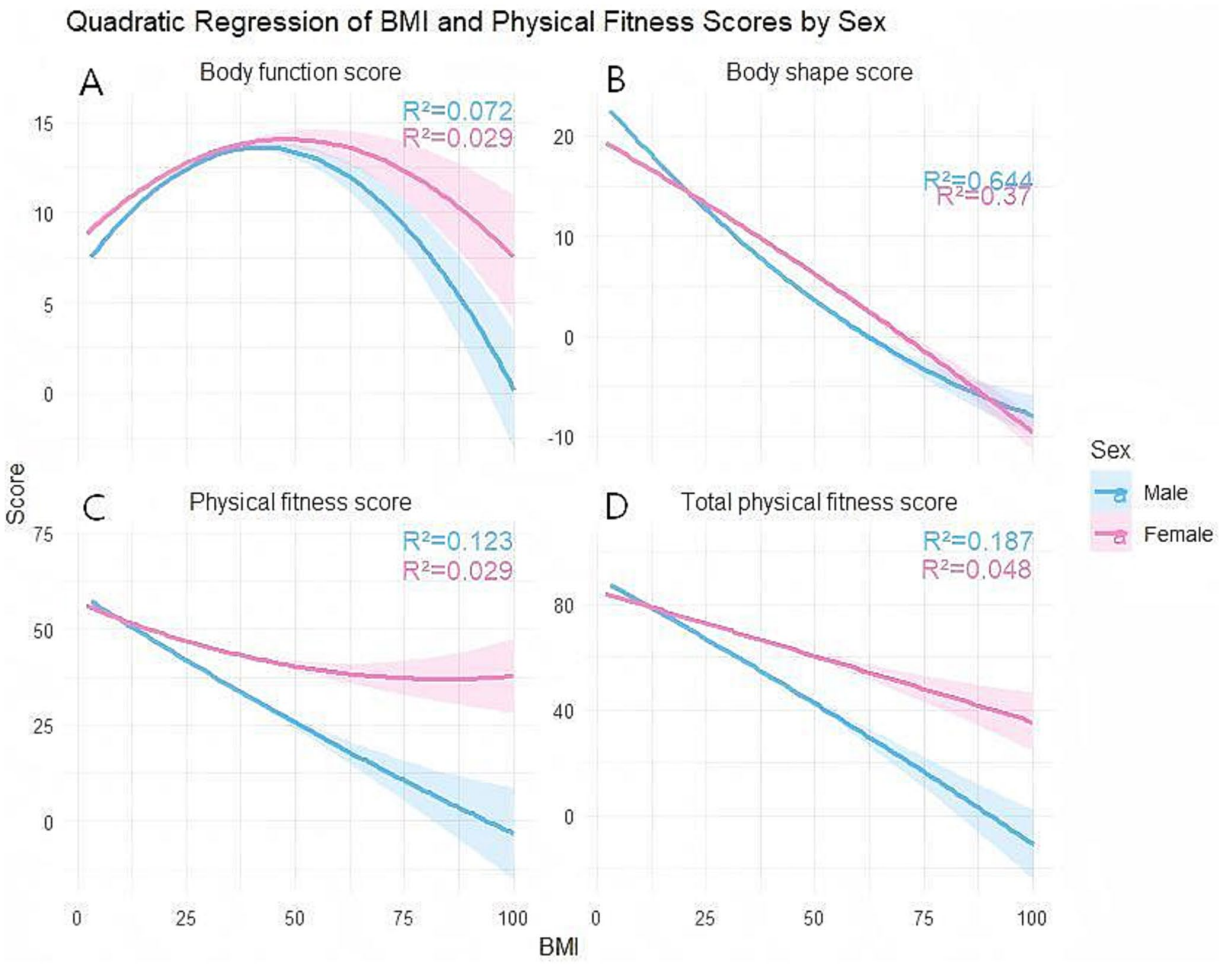
Regression analysis of BMI and physical fitness scores across different BMI categories and genders. **(A)** Regression analysis of physical fitness scores among college students at different BMI levels and of different genders. **(B)** Regression analysis of body shape scores among college students at different BMI levels and of different genders. **(C)** Regression analysis of physical fitness scores among college students at different BMI levels and of different genders. **(D)** Regression analysis of total fitness test scores among college students at different BMI levels and of different genders.

Figure 4 presents the quadratic regression model for physical function scores (A), physical morphology scores (B), body mass scores (C), and total physical fitness scores (D). The results indicate that the relationship between BMI and physical fitness scores is not a simple linear one, but rather exhibits distinct nonlinear characteristics, with differing relationship patterns across various dimensions of physical fitness. Both male and female students’ physical fitness scores exhibit an inverted U-shaped relationship, meaning that as BMI increases, fitness scores initially rise before declining. This suggests the existence of a relatively optimal BMI range where fitness levels peak. BMI showed a significant overall negative correlation with body shape scores, and the quadratic term did not alter its monotonically decreasing trend. This indicates that increased BMI—particularly in overweight or obese individuals—significantly lowers body shape evaluations. This relationship is more pronounced among men, suggesting that BMI has greater explanatory power for men’s body shape evaluations. Physical fitness scores and overall fitness scores both decline with increasing BMI, with a steeper decline observed in males, indicating that elevated BMI exerts a significant inhibitory effect on athletic performance and overall physical fitness levels. The relatively flatter curve for women indicates that BMI has a comparatively weaker negative impact on their physical fitness levels.

Beyond statistical significance, the coefficient of determination (*R*^2^) was used to evaluate the practical explanatory power of BMI on physical fitness outcomes. The regression curve and coefficient of determination (*R*^2^) indicate that the relationship between BMI and physical fitness scores exhibits significant differences between genders. The *R*^2^ values for the male model were generally higher than those for the female model, particularly in body shape scores (male *R*^2^ ≈ 0.644, female *R*^2^ ≈ 0.37) and total physical fitness scores (male *R*^2^ ≈ 0.187, female *R*^2^ ≈ 0.048). This indicates that BMI explains physical fitness-related indicators more strongly in males, while female physical fitness may be influenced more significantly by factors beyond BMI, such as body composition distribution and exercise participation behavior. Male physical fitness scores decline more rapidly with increasing BMI, suggesting that overweight and obesity exert a more direct negative impact on male physical performance. In contrast, females exhibit a certain buffering effect, potentially related to fat distribution characteristics and the structure of physical fitness scoring. The *R*^2^ values of the regression models across various dimensions generally fall within the low to moderate range, indicating that while BMI is an important factor influencing physical fitness, it is not the sole determinant. Physical fitness levels may also be influenced by the combined effects of multiple variables, including training levels, lifestyle, genetic background, and psychological factors.

## 4 Discussion

This study systematically analyzed the association between BMI and multidimensional physical fitness indicators based on physical health monitoring data from 28,861 college students at a university in Yangzhou, China. Research findings indicate that the relationship between BMI and physical fitness levels among college students is not a simple linear correlation, but rather exhibits a nonlinear pattern with the normal weight range being the optimal zone. At the same time, BMI exhibits significant heterogeneity in its impact across different physical fitness test dimensions and between genders.

Research indicates that BMI exhibits a stable inverted U-shaped or monotonically decreasing relationship with overall fitness levels and most physical fitness indicators. Specifically, students of normal weight demonstrate optimal performance in core fitness metrics such as speed, endurance, explosive power, and muscular endurance, whereas overweight and obese conditions are significantly associated with diminished physical fitness levels. This aligns with previous research findings on Chinese university students, indicating that those with normal BMI fall within the normal weight range and perform better in various physical fitness tests (32–35). Normal BMI is generally associated with a more favorable body composition, characterized by higher relative lean mass and lower fat mass, which supports strength production, metabolic efficiency, and exercise economy (36). In overweight and obese states, excess adipose tissue not only increases mechanical load during exercise but may also be associated with impaired endurance and strength fitness, potentially through pathways such as reduced insulin sensitivity, altered mitochondrial function, and chronic low-grade inflammation (37, 38). Underweight students may experience insufficient muscle mass and limited energy reserves, making them more prone to functional limitations during high-intensity or sustained physical activity (39, 40). This underscores the importance of maintaining a healthy BMI range for sustaining functional health.

Unlike most physical fitness indicators, lung capacity is positively correlated with BMI, with overweight and obese students exhibiting higher values than those who are underweight (41). This finding underscores the heterogeneous effects of BMI across physiological systems. Lung capacity primarily reflects static ventilatory capacity and is influenced by body size, thoracic dimensions, and respiratory muscle strength, rather than dynamic cardiorespiratory endurance during exercise (42). Higher lung capacity in individuals with elevated BMI may reflect a larger thoracic cavity, increased respiratory muscle workload, or greater absolute body size, all of which can influence measurements of static lung volumes (35, 43, 44). However, students with higher BMIs consistently performed worse in speed, endurance, and strength-related tasks. A study by Dewi et al. (45) indicates that increased fat mass is associated with declining physical health. A better indicator for assessing physical fitness in obese individuals is the percentage of fat mass rather than BMI (45). This discrepancy reflects that isolated measurements may not fully capture functional fitness, and supports the use of multidimensional fitness assessments in population-based health evaluations.

Significant gender differences were also observed in the relationship between BMI and physical fitness. Overall, males outperform females in metrics such as speed, explosive power, strength, and lung capacity, while females hold a distinct advantage in flexibility. This aligns with findings from Allison et al. (46). More importantly, the negative association between increased BMI and physical fitness appears stronger in men than in women. However, this gender disparity should be interpreted with caution, as it may partly reflect differences in the structure and scoring systems of physical fitness tests between men and women rather than purely biological differences (47, 48). Regression analysis indicates that men’s physical fitness scores decline more steeply with increasing BMI, and BMI explains a greater proportion of variation in men’s physical fitness indicators overall than in women. This phenomenon may be related to the fact that physical fitness tests for men place greater emphasis on relative strength and load-bearing capacity (32, 49, 50). In the Chinese national physical fitness test, pull-ups and long-distance endurance runs require male participants to repeatedly perform weight-bearing or lifting movements equivalent to their own body weight. Consequently, excess body weight may impose disproportionate mechanical stress on male participants, thereby amplifying the correlation between BMI and physical fitness levels (51, 52). In contrast, women’s physical fitness performance shows relatively lower sensitivity to BMI, which may indicate that BMI is a less sensitive proxy for functional fitness under the current female-specific testing structure, where fewer items directly penalize excess body mass (50, 53, 54). These findings indicate that BMI-based evaluations may be less informative for explaining fitness variability among female students.

The findings of this study have direct implications for health promotion and physical education programs in higher education. The nonlinear association between BMI and physical fitness highlights the importance of maintaining a normal weight range, suggesting that university health interventions should address both overweight/obesity and underweight status rather than focusing solely on obesity prevention (55, 56). The heterogeneous effects of BMI across fitness components indicate that BMI alone is insufficient to capture students’ functional health (57, 58). Routine health monitoring in universities should therefore combine BMI with multidimensional physical fitness assessments to enable more accurate identification of fitness-related health risks and guide targeted interventions. In addition, the observed gender differences suggest that health promotion strategies should be gender-sensitive (59). For male students, excess body weight appears to impose greater limitations on relative strength and endurance, underscoring the importance of weight management and load-bearing capacity training (45). For female students, physical fitness may be less directly constrained by BMI, indicating the need for interventions that emphasize physical activity participation, muscle strengthening, and exercise quality (60).

This cross-sectional study has several limitations that should be acknowledged. First, the cross-sectional design inherently precludes causal inference. Although an association between BMI and physical fitness indicators has been observed, these findings should be interpreted as correlational rather than causal. Future longitudinal research employing repeated measurements of BMI and physical fitness indicators across multiple time points is warranted to clarify temporal ordering, developmental trajectories, and the dynamic effects of BMI changes on physical fitness over time. Second, BMI was used as the primary anthropometric indicator in this study. While BMI is widely applied in population-based research due to its simplicity and feasibility, it does not differentiate between fat mass and lean mass. This limitation may be particularly relevant for physically active students and male participants, for whom higher BMI values may reflect greater muscle mass rather than excess adiposity. Consequently, the use of BMI alone may lead to misclassification of body composition and potentially obscure more nuanced associations between adiposity and physical fitness. Future studies should incorporate more precise body composition measures, such as body fat percentage, waist circumference, or dual-energy X-ray absorptiometry, to improve the accuracy of health risk assessment. Third, this study did not systematically collect information on several potential confounding factors, including dietary habits, physical activity intensity outside of mandatory testing, sleep patterns, and psychological stress. These unmeasured confounding variables may have influenced both BMI and physical fitness outcomes, leading to residual confounding that could not be fully controlled in the analysis. Future research should integrate comprehensive lifestyle and psychosocial assessments to better account for these factors. Furthermore, Physical fitness scores are determined based on the national grading system, which employs distinct scoring criteria for first/s-year students and third/fourth-year students. While these standards aim to reflect developmental differences across grade levels, applying grade-specific criteria when comparing physical fitness outcomes across the entire sample may introduce additional variability. This potential source of measurement heterogeneity should be considered. Finally, the study sample was drawn from a single university, which limits the generalizability of the results. Regional, institutional, and cultural characteristics, as well as potential self-selection bias among students with higher health awareness, may restrict the applicability of the findings to the broader population of Chinese college students. Consequently, future research should conduct multi-center studies covering different regions and types of universities to enhance external validity.

## 5 Conclusion

This study identified a significant association between BMI and multiple dimensions of physical fitness among Chinese college students, with evident heterogeneity by gender. Physical fitness performance was highest among students within the normal BMI range, while both underweight and overweight/obese BMI categories were associated with lower performance across several fitness indicators, including speed, endurance, strength, and overall fitness scores.

The relationship between BMI and overall physical fitness exhibited a nonlinear, inverted U-shaped pattern, indicating that deviations from the normal BMI range—either lower or higher—were associated with reduced physical fitness levels. In addition, the strength of the association between BMI and physical fitness differed by gender, with stronger negative associations observed among male students compared to female students.

Based on large-sample observational data, this study adds to the existing evidence on the relationship between BMI and multidimensional physical fitness in the college student population. The findings highlight the importance of considering nonlinear patterns and gender differences when examining BMI–fitness associations. However, given the cross-sectional nature of the data, the observed relationships should be interpreted as associative rather than causal. Future intervention-based studies, such as randomized or quasi-experimental designs targeting BMI management, physical activity, or health-related behaviors, are needed to test whether modifying these factors leads to subsequent improvements in physical fitness. Together, longitudinal and intervention-based approaches would strengthen causal inference and facilitate the translation of the present findings into evidence-based health promotion strategies for college students.

## Data Availability

The original contributions presented in the study are included in the article/supplementary material, further inquiries can be directed to the corresponding author.
